# The clinical consequences of burst abdomen after emergency midline laparotomy: a prospective, observational cohort study

**DOI:** 10.1007/s10029-024-03104-x

**Published:** 2024-07-20

**Authors:** Madeline Kvist, Thomas Korgaard Jensen, Christian Snitkjær, Jakob Burcharth

**Affiliations:** 1 Department of Gastrointestinal and Hepatic Diseases, Copenhagen University Hospital – Herlev and Gentofte, Borgmester Ib Juuls Vej 1, 2730 Herlev, Denmark; 2 Emergency Surgery Research Group Copenhagen (EMERGE Cph), Copenhagen University Hospital - Herlev and Gentofte Herlev, Herlev, Denmark

**Keywords:** Laparotomy, Burst abdomen, Wound healing, Complications, Morbidity

## Abstract

**Purpose:**

The emergency midline laparotomy is a commonly performed procedure with a burst abdomen being a critical surgical complication requiring further emergency surgery. This study aimed to investigate the clinical outcomes of patients with burst abdomen after emergency midline laparotomy.

**Methods:**

A single-center, prospective, observational cohort study of patients undergoing emergency midline laparotomy during a two-year period was done. Abdominal wall closure followed a standardized technique using monofilament, slowly absorbable suture in a continuous suturing technique with a suture-to-wound ratio of at least 4:1. Treatment of burst abdomen was surgical. Data, including intra-hospital postoperative complications, were collected and registered chronologically based on journal entries. The primary outcome was to describe postoperative complications, length of stay, and the overall morbidity based on the Comprehensive Complication Index (CCI), stratified between patients who did and did not suffer from a burst abdomen during admission.

**Results:**

A total of 543 patients were included in the final cohort, including 24 patients with burst abdomen during admission. The incidence of burst abdomen after emergency midline laparotomy was 4.4%. Patients with a burst abdomen had a higher total amount of complications per patient (median of 3, IQR 1.3–5.8 vs. median of 1, IQR 0.0–3.0; p = 0.001) and a significantly higher CCI (median of 53.0, IQR 40.3–94.8 vs. median of 21.0, IQR 0.0–42.0; p =  < 0.001).

**Conclusion:**

Patients with burst abdomen had an increased risk of postoperative complications during admission as well as a longer and more complicated admission with multiple non-surgical complications.

**Supplementary Information:**

The online version contains supplementary material available at 10.1007/s10029-024-03104-x.

## Introduction

Each year, approximately one out of 1100 of the population undergo emergency laparotomy, often due to high-risk surgical conditions such as bowel obstruction, gastrointestinal perforation, mesenteric ischemia, or other high-risk surgical conditions [1, 2]. Due to the need for maximum visibility of the peritoneal cavity, midline laparotomy is most often the strategy of choice in the emergency setting. However, emergency laparotomy is also associated with high rates of mortality, morbidity, and postoperative complications [2–4].

Burst abdomen is defined as a deep wound rupture of the sutured abdominal midline aponeurosis. The rate of burst abdomen is reported as 3.8–28.0% in emergency surgery [3, 5, 6] and a lower rate of 0.2–5.0% in elective surgery [7–10]. It is regularly described as a severe complication due to increased mortality [11–13], low quality of life [14] and decreased survival [10]. Multiple studies have focused on the identification of patients at higher risk of burst abdomen, finding male sex, old age, frailty, and comorbidities such as obesity, tobacco use, alcohol abuse, and hypertension to be risk factors [3, 4, 6, 7, 11, 15–17]. Emergency surgery and closure techniques are known as non-patient-related risk factors [3, 4, 6, 7, 11, 15, 17–22], but newer studies have also focused on the quality of the midline aponeurosis [23]. Despite a greater focus on defining patients at risk and decreasing the rate of burst abdomen, no studies have elaborated upon why exactly burst abdomen is dangerous or what organ-specific complications lead to the more protracted and complicated course of admission for patients with burst abdomen.

We hypothesized that patients with a burst abdomen have higher rates of mortality and morbidity due to a higher tendency for complications during admission as well as a different pattern of organ-specific complications compared to patients without a burst abdomen. The aim of this prospective, observational cohort study was to investigate the clinical consequences of a burst abdomen after emergency midline laparotomy and fill a gap in the current literature.

## Methods

This study is a single-center, prospective, observational cohort study of patients undergoing emergency midline laparotomy at Copenhagen University Hospital, Herlev, in the years 2021 and 2022. Copenhagen University Hospital, Herlev, is a specialized tertiary university hospital with an emergency catchment area of 465.000 patients. The surgical department performs approximately 450 emergency midline laparotomies and an estimated 100 elective midline laparotomies annually, as the majority of elective surgery is planned as minimally invasive surgery. An emergency laparotomy bundle-of-care approach was implemented in the department in 2017 for patients presenting with acute abdominal pain and clinical suspicion of high-risk surgical conditions [3, 4]. The bundle includes preoperative standardized protocols, a standardized intraoperative approach, and, since 2021, a dedicated and highly specialized ward for postoperative care [24–26].

The study was approved by the Capital Region of Demark (P-2020–1166, R-21038079) and the Danish Data Protection Agency (P-2021–431). The project did not qualify for ethics approval by Danish law, nor required informed consent, due to the observational nature of the study with no intervention or randomization carried out. The study was conducted and reported by The Reporting of Observational Studies in Epidemiology (STROBE) statement [27].

### Abdominal wall closure and strategy of burst abdomen

In our department, closure of the abdominal wall after midline laparotomy has followed a standardized intraoperative strategy since June 2017 and has reduced the rate of burst abdomen since implementation [3, 4, 28]. Primary midline abdominal wall closure is carried out based on international recommendations, utilizing a slow-absorbable, monofilamentous suture in a continuous manner, taking small bites of 5–9 mm aponeurosis and small steps of 5 mm between each step securing a suture-to-wound ratio of at least 4:1 [3]. The abdominal wall closure technique is documented in the patient journal as a standard. Treatment of a burst abdomen is always intended surgically with the aim of early fascial closure, preferably at the first re-operation. A slow absorbable, monofilamenteous suture is used in a continuous manner taking large mass-closure bites of 2–3 cm with small steps of 5 mm between stitches [4]. Negative pressure wound therapy with or without mesh-mediated fascial traction is used in cases with unobtainable fascial closure and only until fascial closure is possible [4].

### Data sources and management

Data were collected via the electronic journal system and included demographic data, American Society of Anaesthesiologists (ASA) score, the World Health Organization/Eastern Cooperative Oncology Group (WHO/ECOG) Performance Status [29], body mass index (BMI), pre-existing comorbidities, risk-factors such as tobacco- and alcohol habits, intra-hospital postoperative complications, length-of-stay, stay in the intensive care unit, and 30- and 90-day mortality. All complications were registered chronologically based on journal entries. Complications were placed within predefined subgroups based upon organ systems being surgical, wound-related, infectious, cerebral, pulmonary, cardiac, thromboembolic, renal, or other. All subgroups and individual complications are also described further in Supplementary Table 1. Complications were scored according to the Clavien-Dindo Classification, and a total score of the overall morbidity was conducted by the Comprehensive Complication Index [30].

Storage and management were performed using the REDCap (Research Electronic Data Capture) platform hosted at our department. The platform is a secure, web-based software designed to support data collection, storage, and management of research studies [31].

### Participants and study size

Patients were evaluated for inclusion during a consecutive inclusion period from January 1st, 2021, to December 31st, 2022. Hereby expecting a study size of approximately 500–600 patients based on the annual normal in our hospital. Adult patients (≥ 18 years) undergoing emergency midline laparotomy during the study period were evaluated for inclusion. Patients were identified by reviewing the surgical log and the patient journals via our journal system (EPIC Hyperspace, Epic Systems Corp 2020). Patients who were underage (< 18 years) or had no Danish social security number were excluded. Patients in the group with burst abdomen were identified in the surgical log in case of surgical treatment of burst abdomen (after index emergency or emergent re-operation) or identified via the patient journal due to experiencing burst abdomen as a postoperative complication. Due to this design, burst abdomen was excluded as a complication in further analysis of complications between the groups.

### Outcome measures

The primary outcome was to describe postoperative complications as well as overall morbidity based on the Comprehensive Complication Index (CCI) in our cohort, stratified between patients who did and did not suffer from a burst abdomen during admission. We aimed to describe the organ-specific complications in each group. Secondary outcomes included length-of-stay, stay in the intensive care unit during admission, days from primary operation to burst abdomen, and 30- and 90-day mortality.

### Statistical analysis

Categorical data were presented as frequencies and percentages. To investigate differences in categorical variables, Pearson’s chi-squared test or two-tailed Fisher’s exact test was used depending on cell count. Continuous data were expressed as mean (standard deviation, SD) and median (interquartile range, IQR). The distribution of continuous data was assessed by visual inspection of histograms and QQ plots. Groups were compared with an unpaired t-test or a Mann–Whitney U test depending on the normality of data distribution. Missing data were reported (Table 1) and only occurred in the baseline data; BMI, Performance Status, tobacco- and alcohol habits. All statistical tests were two-sided. P values ≤ 0.05 were considered statistically significant. Statistical analyses were performed using IBM SPSS statistics for Windows Ver. 29.0.1.0.

**Table 1:** Baseline demographics

All *n* = 543	Group with burst abdomen *n* = 24 (%)		Group without burst abdomen *n* = 519 (%)		*P*-value
Sex, male	18	(75.0)	255	(49.1)	**0.020**
Age, years					
Median (IQR)	71	(58–78)	72	(61–80)	0.289
Body mass index					
Median (IQR) Obesity^*^ ≥ 30 kg/m^2^ Missing data	23.7 2 0	(21.1–27.3) (8.3) (0.0)	24.0 78 6	(21.1–27.6) (15.0) (1.2)	0.927 0.557
ASA score					
III–IV I–II	5 19	(20.8) (79.2)	37 482	(7.1) (92.9)	**0.031**
WHO performance status					
3–4 0–2 Missing data	4 20 0	(16.7) (83.3) (0.0)	57 459 3	(11.0) (88.4) (0.6)	0.335
Comorbidities					
Active malignancy Apoplexy Diabetes Hypertension Atrial fibrillation Chronic ischaemic heart disease Chronic obstructive pulmonary disease Chronic renal disease Liver cirrhosis	9 0 3 8 0 1 1 3 2	(37.5) (0.0) (12.5) (33.3) (0.0) (4.2) (4.2) (12.5) (8.3)	121 37 57 183 68 33 49 16 12	(23.3) (7.1) (11.0) (35.3) (13.1) (6.4) (9.4) (3.1) (2.3)	0.139 0.394 0.740 1.000 0.059 1.000 0.715 **0.046** 0.123
Tobacco use					
Active smoker Non-smoker^**^ Missing data	7 17 0	(29.2) (70.8) (0.0)	97 420 2	(18.7) (81.0) (0.4)	0.235
Alcohol habits					
> Upper limit^+^ < Upper limit^+^ Missing data	7 17 0	(29.1) (70.9) (0.0)	178 337 4	(34.3) (64.9) (0.8)	0.127

*IQR* Interquartile Range, *ASA* American Association of Anesthesiologists, *WHO* World Health Organization

^*^ Obesity defined as Body Mass Index ≥ 30 kg/m^2^ as defined by the Danish Health Authorities

^**^ Defined as never been a smoker or former smoker with no smoking for the last 8 weeks for longer

^+^ Defined by newest recommendations by the Danish Health Authorities with an upper limit for alcohol consumption of 10 units per week for adults (≥ 18 years), irrespective of sex

## Results

**Fig. 1:**
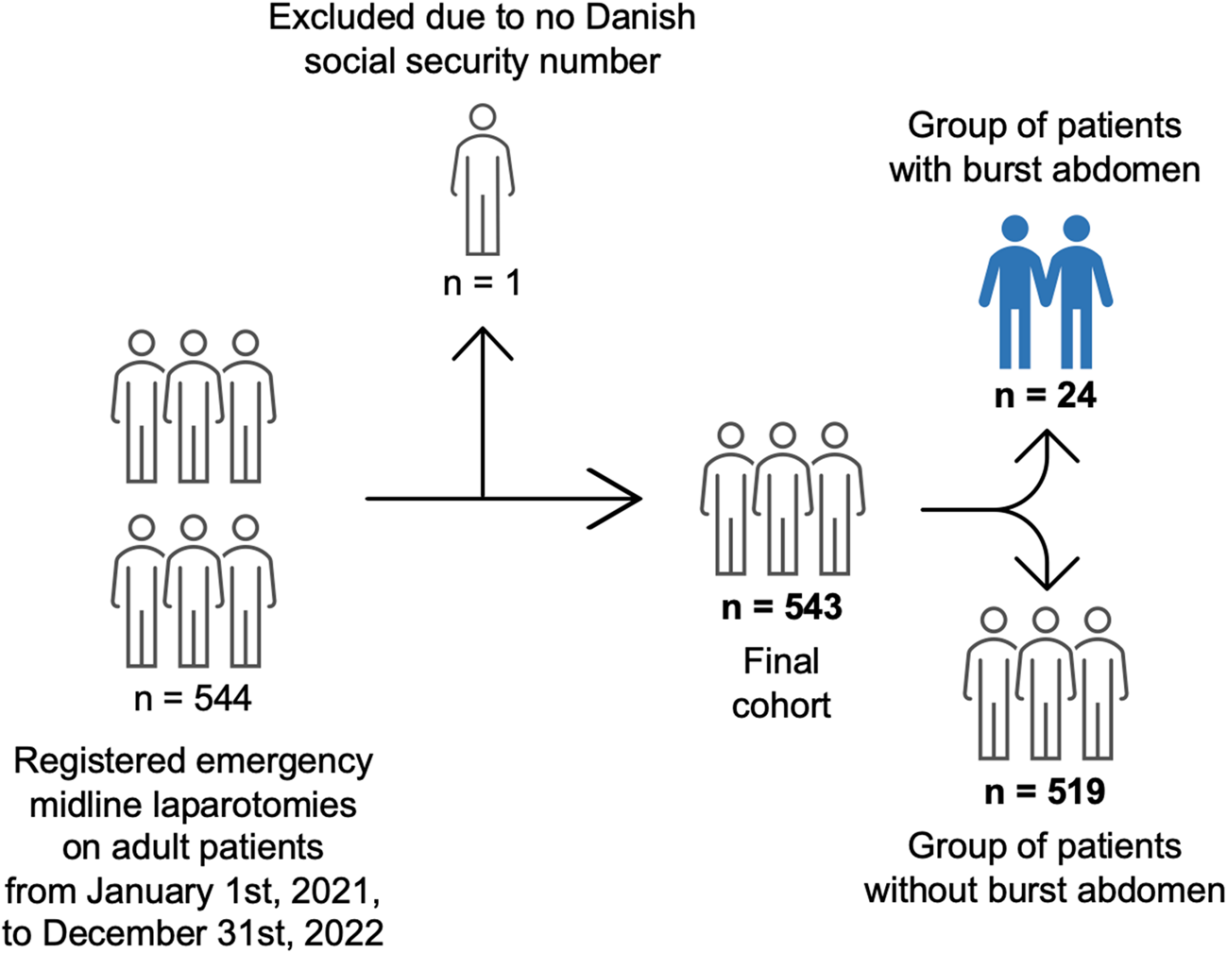
Flowchart of the study population

During the two-year study period, a total of 544 adult patients underwent emergency midline laparotomy in our department. One patient was excluded due to not fulfilling the inclusion criteria of having a Danish social security number allowing for follow-up. The final cohort consisted of 543 individual patients who were eligible for further analyses (Fig. 1). In the cohort, a total of 24 patients (4.4% of 543 patients) experienced a burst abdomen after emergency midline laparotomy. Eighteen (3.3%) patients had burst abdomen after an index emergency laparotomy and six (1.1%) patients from emergent re-operation after elective surgery.

The basic characteristics of the study population are outlined in Table 1. Baseline demographic variables were mostly comparable between the two groups, i.e. patients with a burst abdomen and patients without a burst abdomen, except for male sex being predominant among patients suffering from burst abdomen (18 of 24 patients, 72.0% vs. 255 of 519 patients, 49.1%; p = 0.020) as well as more often demonstrating a high ASA score of III-IV (5 of 24, 20.8% vs. 37 of 519, 7.1%; p = 0.031). We also found slight differences in atrial fibrillation being more common in the group without a burst abdomen (68 of 519, 13.1% vs. 0 of 24, 0.0%; p = 0.059) and chronic renal failure being more common in the group with a burst abdomen (3 of 24, 12.5% vs. 16 of 519, 3.1%; p = 0.046). There were no patients with missing data in the group with a burst abdomen. The group without a burst abdomen had minimal missing data of BMI, WHO Performance Status, tobacco use, and alcohol habits of 1.2%, 0.6%, 0.4%, and 0.8%, respectively.

Regarding the 24 patients with a burst abdomen, a total of 21 (87.5%) had a successful closure of the abdominal wall. Of the remaining three patients, one patient died before closure and two were not able to undergo further surgery and a conservative strategy was chosen instead. We found that the primary closure technique followed the standardized method in 23 (95.8%) patients and had a median suture-to-wound-ratio of 5:1 (minimum 4:1 and maximum 6:1). The standardized regimen with mass-closure technique was followed in 17 (70.9%) patients and a median suture-to-wound ratio was found to be 15:1 (minimum 10:1 and maximum 22:1). Data on abdominal wall closure for the patients with burst abdomen can be seen in Table 2.

**Table 2:** Abdominal wall closure of patients with burst abdomen

Group with Burst Abdomen, *n* = 24 (%)		
Primary closure technique		
Small bites, small steps^*^	23	(95.8)
Suture-to-wound ratio, median (range)	5.0	(4.0–6.2)
Treatment of burst abdomen		
Mass-closure^**^	17	(70.8)
Suture-to-wound ratio, median (range)	15.0	(10.0–22.0)
Use of permanent mesh	6	(25.0)
Use of intraabdominal VAC	6	(25.0)
Subcutaneous VAC	12	(50.0)
Patients with redehiscence	2	(8.3)
Patients with successful closure	21	(87.5)

*VAC* vacuum assisted closure

^*^ Monofilament, slowly absorbable suture in a continuous suturing technique with a suture-to-wound ratio of at least 4:1 by taking small bites of 5 mm of the fascia and small steps of 5 mm between each stitch with self-locking knots

^**^ Monofilament, slowly absorbable suture in a continuous suturing technique with a suture-to-wound ratio of at least 10:1 by taking big bites of 3 cm of the fascia and small steps of 5 mm between each stitch with self-locking knots

**Fig. 2:**
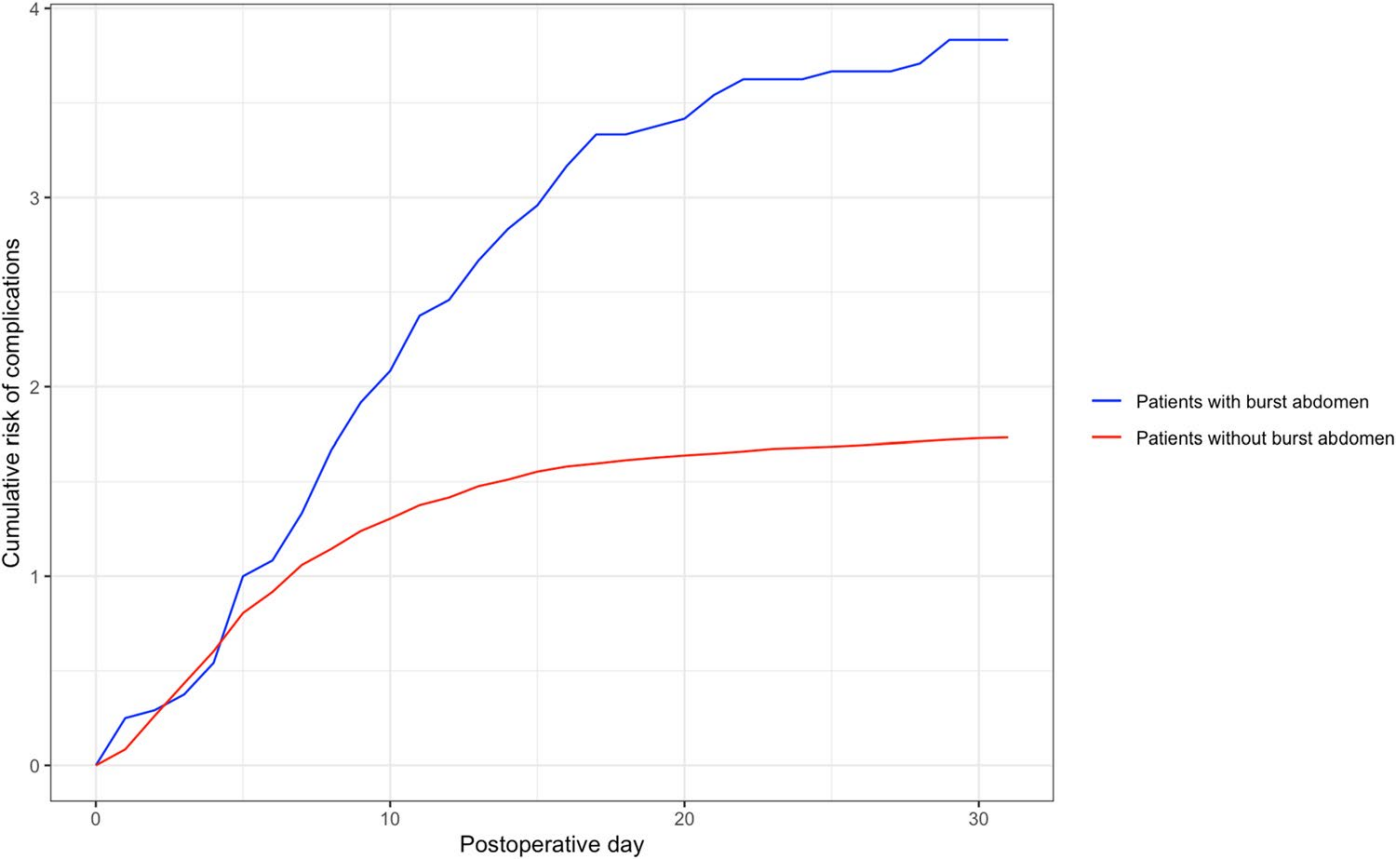
Cumulative risk of complications in each group. Chart of the cumulative risk of complications for the group with burst abdomen (blue) and the rest of the cohort without burst abdomen (red). The x-axis shows postoperative days from day of surgery (day 0) to the 30^th^ postoperative day. The y-axis shows the cumulative risk of complications as a ratio of each group (24 patients with burst abdomen and 519 patients without)

**Fig. 3:**
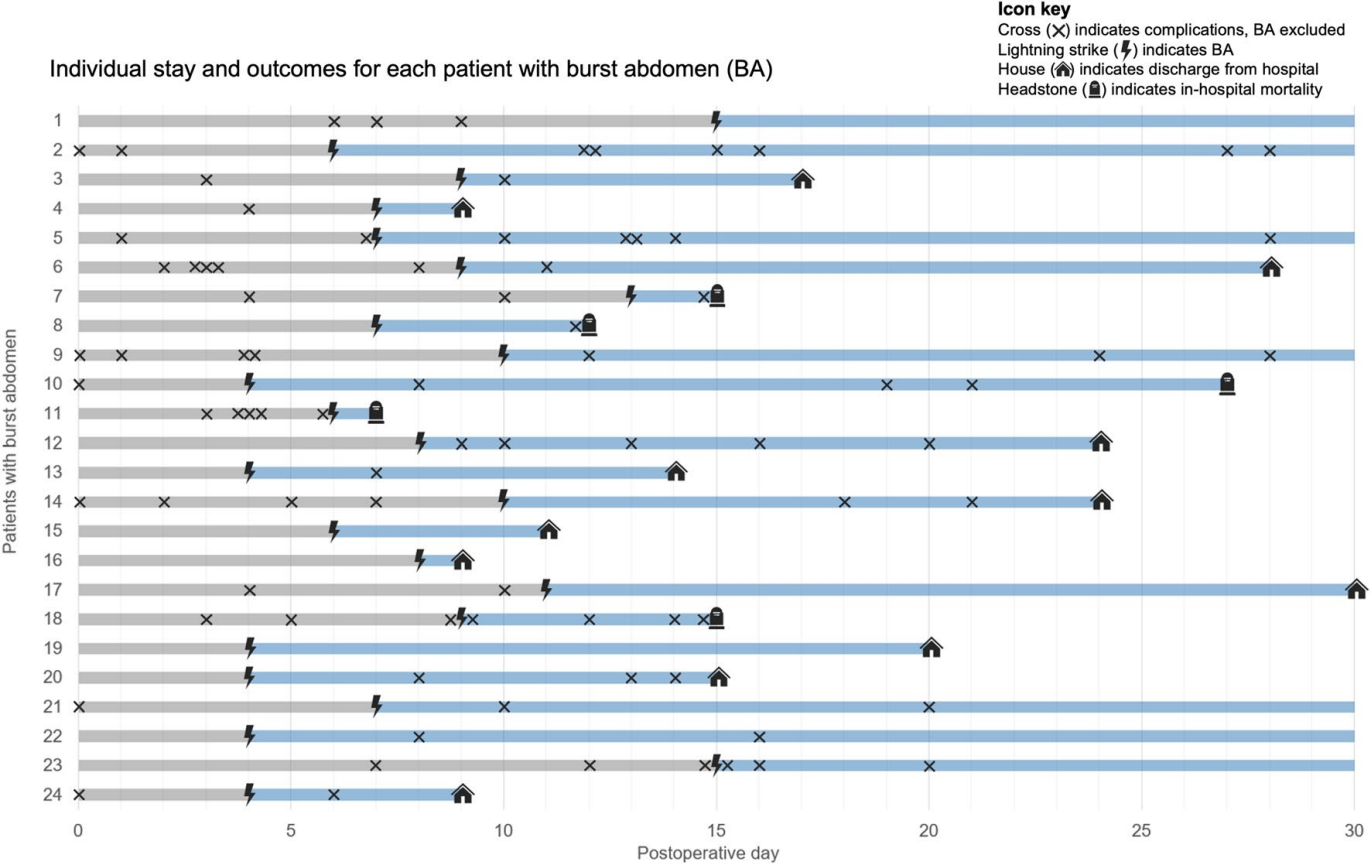
Schematic presentation of outcomes for patients with burst abdomen. Illustration of the individual stay and outcomes for all 24 patients with burst abdomen. The x-axis shows postoperative days from day of surgery (day 0) to the 30^th^ postoperative day. The y-axis shows the individual patients from 1 to 24 in chronological order according to day of surgery. Icons illustrate outcomes during stay with cross indicating complications, lightning strike indicating day of burst abdomen, house indicating discharge from hospital and headstone indicating in-hospital mortality

An overview of clinical outcomes is listed in Table 3. In the group of patients with a burst abdomen, we found that 21 of 24 patients (87.5%) had postoperative complications other than burst abdomen during admission, which was seen to be an increased tendency compared to the 358 of 519 patients in the rest of the cohort (69.0%). Yet the data was not significant (p = 0.067). Patients with a burst abdomen had a higher total amount of complications per patient (median of 3 and IQR of 1.3–5.8 vs. median of 1 and IQR of 0.0–3.0; p = 0.001). Furthermore, the group with a burst abdomen also had a significantly higher Comprehensive Complication Index (CCI) than the rest of the cohort without a burst abdomen (median of 53.0 and IQR of 40.3–94.8 vs. median of 21.0 and IQR of 0.0–42.0; p =  < 0.001) referring to an overall greater score of postoperative morbidity. The cumulative risk of complications was generally higher in the group with a burst abdomen, especially after the fifth postoperative day – and patients with a burst abdomen were found to have more complications on the eight postoperative day than the patients without a burst abdomen had on the 30th postoperative day (Fig. 2). For the group of patients with burst abdomen, we stratified the number and type of complications before and after burst abdomen, which is shown in Table 4. The median number of days from index surgery to burst abdomen was seven days (IQR 4.5–9.8 days). The patients tended to have approximately the same amount of overall complications before and after burst abdomen (median of 1 and IQR of 0.0–2.8 vs. median of 1 and IQR of 0.0–3.0). However, a difference was seen regarding CCI before and after burst abdomen, with an overall greater score of morbidity after burst abdomen (median 10.5 and IQR of 0.0–39.4 before vs., median of 41.6 and IQR of 0.0–90.0). Differences before and after burst abdomen were seen regarding some organ-specific complications, with the biggest differences seen in cardiac complications (1 of 24 patients, 4.2%, before and 6 of 24 patients, 25.0%, after). A schematic illustration of stay and outcomes for all patients with a burst abdomen is shown in Fig. 3.

**Table 3:** Overview of clinical outcomes

All *n* = 543	Group with Burst Abdomen *n* = 24 (%)		Group without Burst Abdomen *n* = 519 (%)		*P*-value
Complications^+^					
Any complications	21	(87.5)	358	(69.0)	0.067
Total complications, median (IQR)	3	(1.3–5.8)	1	(0.0–3.0)	**0.001**
Comprehensive Complication Index^*^					
Median (IQR)	53.0	(40.3–94.8)	21.0	(0.0–42.0)	** < 0.001**
Complication type					
Surgical^+^	11	(45.8)	143	(27.6)	0.064
Wound related	9	(37.5)	69	(13.3)	**0.004**
Infectious	6	(25.0)	95	(18.3)	0.421
Cerebral	4	(16.7)	55	(10.6)	0.317
Pulmonary	11	(45.8)	105	(20.2)	**0.005**
Cardiac	7	(29.2)	57	(11.0)	**0.015**
Thromboembolic	1	(4.2)	4	(0.8)	0.203
Renal	8	(33.3)	163	(31.4)	1.000
Other	8	(33.3)	154	(29.7)	0.820
Length-of-stay, days					
Median (IQR)	22.0	(12.5–34.3)	7.0	(5.0–13.0)	** < 0.001**
Stay in ICU during admission	7	(29.2)	93	(17.9)	0.178
Mortality					
30-day mortality	5	(20.8)	59	(11.4)	0.186
90-day mortality	7	(29.2)	93	(17.9)	0.178

*IQR* Interquartile Range, *ICU* Intensive Care Unit

^+^ Complications other than burst abdomen

^*^ Score of the overall morbidity on a scale from 0 to 100, with 0 being no complications and 100 being death. The score is based on the complication grading by the Clavien-Dindo Classification

**Table 4:** Distrubution of complications before and after burst abdomen

Group with Burst Abdomen, *n* = 24 (%)				
Days to BA				
Median (IQR)	7.0		(4.5–9.8)	
	**Complications before BA**		**Complications after BA**	
Total complications				
Median (IQR)	1.0	(0.0–2.8)	1.0	(0.0–3.0)
Comprehensive Complication Index^*^				
Median (IQR)	10.5	(0.0–39.4)	41.6	(0.0–90.0)
Complication type				
Surgical^+^	4	(16.7)	8	(33.3)
Wound related	5	(20.8)	5	(20.8)
Infectious	3	(12.5)	3	(12.5)
Cerebral	2	(8.3)	2	(8.3)
Pulmonary	5	(20.8)	7	(29.2)
Cardiac	1	(4.2)	6	(25.0)
Thromboembolic	0	(0.0)	1	(4.2)
Renal	6	(25.0)	3	(12.5)
Other	5	(20.8)	4	(16.7)

*BA*, Burst abdomen, *IQR* Interquartile Range

^*^ Score of the overall morbidity on a scale from 0 to 100, with 0 being no complications and 100 being death. The score is based on the complication grading by the Clavien-Dindo Classification

^+^ Complications other than burst abdomen

Wound-related complications (9 of 24 patients, 37.5% vs. 69 of 519 patients, 13.3%; p = 0.004), pulmonary complications (11 of 24, 45.8% vs. 105 of 519, 20.2%; p = 0.005) and cardiac complications (7 of 24, 29.2% vs. 57 of 519, 11.0%; p = 0.015) were more common in the group with a burst abdomen compared to the group without. The distribution of organ-specific postoperative complications is listed in Supplementary Table 1. Patients with a burst abdomen suffered more from postoperative wound infection (4 of 24, 16.7% vs. 18 of 519, 3.5%; p = 0.013). Bronchospasm (3 of 24, 12.0% vs. 8 of 519, 1.5%; p = 0.010) and postoperative bowel obstruction occurred more often in patients with a burst abdomen (2 of 24, 8.3% vs. 5 of 519, 1.0%; p = 0.034). Excluding burst abdomen, we found no significant difference in surgical complications between the two groups (11 of 24, 45.8% vs. 143 of 519, 27.6%; p = 0.064).

Regarding postoperative mortality rates, a clinical but not significant difference was observed in patients with burst abdomen having a higher mortality rate at 30 days (5 of 24 patients, 20.8% vs. 59 of 519 patients, 11.4%; p = 0.186) and 90 days (7 of 24, 29.2% vs. 93 of 519, 17.9%; p = 0.178) after index surgery. Patients with a burst abdomen stayed longer before discharge (median stay of 22 days and IQR of 12.5–34.3 vs. median of 7 days and IQR of 5.0–13.0; p =  < 0.001). The rate of admissions to the intensive care unit during admission were comparable between the two groups (7 of 24, 29.2% vs. 93 of 519, 17.9%; p = 0.178).

## Discussion

In this prospective study of 543 patients undergoing emergency midline laparotomy from January 1st, 2021, to December 31st, 2022, we found that patients experiencing the surgical complication burst abdomen had an increased tendency to suffer from further cardiopulmonary and wound-related complications during admission and had an overall higher score of morbidity based on the Comprehensive Complication Index (CCI).

The rate of burst abdomen after index emergency midline laparotomy was 3.3%, which has remained constant since the standardization of our technique for midline abdominal wall closure after emergency surgery [3]. Including six patients suffering from burst abdomen after emergent re-operation, we had a total of 24 patients with burst abdomen in our cohort. This group had a greater total number of complications during admission and a clinically increased risk of complications in general. We found that the cumulative risk of complications was generally higher (as demonstrated in Fig. 2). This has not been previously described in the literature. Regarding organ-specific complications, wound-related and cardiopulmonary complications were notably high among patients suffering from burst abdomen. Surgical site occurrences (i.e. seroma, hematoma, infection, or rupture) are recognized as risk factors for the development of burst abdomen in the literature [7, 11, 13, 15–17, 32], and a higher incidence of these among patients with burst abdomen was somewhat expected. In our study, patients were equally likely to have wound complications before or after a burst abdomen. Furthermore, we found that burst abdomen mostly occurred on the seventh postoperative day, which correlates to earlier studies [33]. The event of a burst abdomen seemed to accelerate the rate of complications with an overall higher score of morbidity. This tendency is also shown in Fig. 2 with the cumulative risk having a steady rise in complications from the fifth to tenth postoperative day.

Closure of the abdominal wall of the patients with burst abdomen was successful in 87.5% of cases. Our standardized method of primary closure was followed in 95.8% of cases. It is known from the literature, that some patients might have an increased risk of burst abdomen prior to surgery. We found that male sex and high ASA scores were more common among patients suffering from burst abdomen compared to the rest of the cohort without burst abdomen. These are known risk factors for burst abdomen [3, 4, 15, 17] and a higher incidence was expected. We did not find any significant difference regarding BMI, age, WHO Performance Status, tobacco use, alcohol habits, or liver cirrhosis in our study. Yet, these have been described as risk factors in both the literature [3, 6, 7, 11, 15–17] and earlier studies from our group [23, 34, 35]. Patients at risk might benefit from an individualized closure technique. In recent years, studies have focused on the use of prophylactic mesh augmentation at the index laparotomy to reduce risk of a burst abdomen [36–38] and incisional hernia [37–43]. Results have been promising, but further studies are needed to investigate possible methods and identify which patients might benefit the most [28, 44]. Additionally, multiple risk factors for development of incisional hernia have been found including male sex, tobacco use, obesity, diabetes etc., but more data is needed [43, 45–47]. Patients with burst abdomen had a longer length of stay compared to the rest of the cohort. We observed no statistically significant variation in 30- or 90-day mortality rates; nevertheless, there was a discernible clinical distinction.

This prospective, observational cohort study does hold some limitations. Due to data selection from a two-year period and burst abdomen being a rare outcome, we do recognize that our sample size of patients with burst abdomen is small, with 24 cases. This, as well as our study being a single-center cohort study, results in some uncertainty regarding underpowering of data for comparisons as well as data being generalizable. Missing data regarding BMI, WHO Performance Status, tobacco use, and alcohol habits were 0.4–1.2%. However, as this is a prospective study conducted in a center serving a larger population in Denmark, we do believe our data represents a general tendency, although further studies with a larger sample size are needed. This study emphasizes a void in the current literature as to why exactly a burst abdomen is dangerous.

In conclusion, patients with burst abdomen have an increased risk of postoperative complications during admission as well as a longer and more complicated stay with multiple non-surgical complications. Further studies, preferably with larger sample sizes, are needed to confirm and elaborate upon our findings.

## Supplementary Information

Below is the link to the electronic supplementary material.

Supplementary file1 (DOCX 20 KB)

## Data Availability

Data are not publicly available due to being sensitive data but can be available in anonymized format on request to the corresponding author.
