# Supplementary material for: The clinical consequences of burst abdomen after emergency midline laparotomy: a prospective, observational cohort study
Source: Hernia. 2024 Jul 20;28(5):1861–70. doi: 10.1007/s10029-024-03104-x (PMC11449993; doi:10.1007/s10029-024-03104-x)
Supplement: Supplementary file 1 — Supplementary file1 (DOCX 20 KB) [file 10029_2024_3104_MOESM1_ESM.docx]

**Supplementary Table 1: Distribution of organ-specific postoperative complications**

| **All   n = 543** | **Group with  Burst Abdomen  n = 24 (%)** | | **Group without Burst Abdomen  n = 519 (%)** | | **P-value** |
| --- | --- | --- | --- | --- | --- |
| **Surgical^*^**  Anastomotic leakage  Reperforation  Visceral ischemia or necrosis  Intraabdominal abscess  Bowel obstruction   Intestinal paralysis > 7 days  Partial obstruction   Intraabdominal bleeding  Gastrointestinal tract bleeding  Other^a^ | 1 2 1 3 2 4 0 1 2 0 | (4.2) (8.3) (4.2) (12.5) (8.3) (16.7) (0.0) (4.2) (8.3) (0.0) | 10 7 9 23 5 66 12 14 16 22 | (1.9) (1.3) (1.7) (4.4) (1.0) (12.7) (2.3) (2.7) (3.1) (4.2) | 0.395 0.056 0.366 0.111 **0.034** 0.534 1.000 0.497 0.186 0.616 |
| **Wound related^*^**  Wound rupture  Wound infection  Wound bleeding  Other^b^ | 3 4 1 3 | (12.5) (16.7) (4.2) (12.5) | 26 18 4 28 | (5.0) (3.5) (0.8) (5.4) | 0.130 **0.013** 0.203 0.151 |
| **Infectious^*^**  Sepsis   Urinary tract infection  Other^c^ | 3 1 3 | (12.5) (4.2) (12.5) | 39 9 56 | (7.5) (1.7) (10.8) | 0.420 0.366 0.737 |
| **Cerebral^*^**  Delirium  Other^d^ | 3 1 | (12.5) (4.2) | 40 16 | (7.7) (3.1) | 0.426 0.542 |
| **Pulmonary^*^**  Respiratory failure  Atelectasis  Pneumonia  Pleural effusion  Bronchospasm  Pulmonary stasis  Other^e^ | 2 0 5 3 3 2 3 | (8.3) (0.0) (20.8) (12.5) (12.5) (8.3) (12.5) | 30 8 48 17 8 10 3 | (5.8) (1.5) (9.2) (3.3) (1.5) (1.9) (0.6) | 0.646 1.000 0.074 0.053 **0.010** 0.094 **0.001** |
| **Cardiac^*^**  Cardiac arrest  New arrhythmia  Other^f^ | 1 3 3 | (4.2) (12.5) (12.5) | 14 35 10 | (2.7) (6.7) (1.9) | 0.497 0.232 **0.016** |
| **Thromboembolic^*^**  Pulmonary embolism  Other^g^ | 1 0 | (4.2) (0.0) | 1 3 | (0.2) (0.6) | 0.087 1.000 |
| **Renal^*^**  Rise in creatnine > 27 μmol/L  Worsening of chronic kidney failure  Overhydration  Electrolyte imbalance  Other^h^ | 4 1 2 2 0 | (16.7) (4.2) (8.3) (8.3) (0.0) | 46 7 37 90 11 | (8.9) (1.3) (7.1) (17.3) (2.1) | 0.264 0.305 0.688 0.402 1.000 |
| **Other^*^**  Parenteral nutrition  Hepatic encephalopathy  Transfusion  Other^i^ | 4 0 3 3 | (16.7) (0.0) (12.5) (12.5) | 33 5 41 109 | (6.4) (1.0) (7.9) (21.0) | 0.072 1.000 0.432 0.441 |
| ^*^ Complications are recorded as events, meaning patients could experience the same complication multiple times during admission. ^a^ Intraabdominal edema or hematoma, ascites, unsuspected amount of pain, second look operation. ^b^ Stoma complication, wound hematoma, serous drainage, enterocutaneous fitula. ^c^ Intestinal infection, oral candida, unknown focus of infection, Covid-19. ^d^ Postoperative cognitive decline, depression, anxiety, neuropathy, seizure. ^e^ Pneumothorax, lung abscess, empyema. ^f^ Acute myocardial infarction, myocardial infarction after non-cardiac surgery, acute heart failure, worsening of cardiac insufficiency, dysregulated arrhythmia, acute hypertension, cardiac hypertrophy. ^g^ Arterial abdominal embolism, embolism in v. jugularis. ^h^ Dialysis, urinary retention, dehydration. ^I^ Worsening of chronic wound, high output, dyspepsia, palliative care chosen by patient, skin reaction, hypotension, pain, dysregulated diabetes mellitus. | | | | | |
